# Epigenetic regulation of bladder cancer in the context of aging

**DOI:** 10.3389/fphar.2025.1617452

**Published:** 2025-08-21

**Authors:** Xuewei Liu, Guofeng Ding, Yifan Liu, Xiaoli Yan, Yan Zhao, Hailin Lv, Xiaojuan Xu

**Affiliations:** ^1^ Academy of Medical Engineering and Translational Medicine, Tianjin University, Tianjin, China; ^2^ Stem Cell Research Center, Department of Pathology and Pathophysiology, School of Medicine, Tongji University, Shanghai, China; ^3^ Department of Urology, Tongji Hospital, School of Medicine, Tongji University, Shanghai, China; ^4^ Teaching Laboratory Center, School of Medicine, Tongji University, Shanghai, China; ^5^ Department of Nephrology, Qilu Hospital of Shandong University (Qingdao), Qingdao, Shandong, China

**Keywords:** bladder cancer, aging, epigenetics, DNA methylation, histone modifications, non-coding RNAs, immunosenescence

## Abstract

Bladder cancer (BC) is a disease that predominantly affects older adults, with aging playing a critical role in its onset and progression. Age-associated phenomena, including immunosenescence and chronic inflammation, form a pro-tumor milieu, while genomic instability and epigenetic drift further increase cancer risk. The review highlights the dual role of DNA methylation in BC: global hypomethylation can activate transposable elements and oncogenes, whereas focal hypermethylation silences tumor-suppressor genes like CDKN2A, especially detrimental in older tissues that rely on these genes for senescence control. In parallel, frequent mutations in chromatin modifiers (e.g., KDM6A, KMT2D) and overexpression of histone-modifying enzymes (e.g., EZH2) alter the tumor epigenome to promote immune evasion and tumor aggressiveness. At the non-coding RNA level, dysregulated microRNAs (miRNAs) and long non-coding RNAs (lncRNAs) in BC contribute to aberrant proliferation, metastatic potential, and immune suppression, with aging-associated declines in miRNA processing further exacerbating these effects. Collectively, the accumulation of epigenetic alterations in older patients appears to facilitate both tumor progression and resistance to therapy. Looking forward, epigenetic biomarkers may improve early detection and risk stratification. Furthermore, “epigenetic therapies,” such as DNA methyltransferase inhibitors (DNMTi), EZH2 inhibitors (EZH2i), or histone deacetylases inhibitors (HDACi), hold promise to restore tumor-suppressor function and enhance immunogenicity, offering an attractive avenue for improving outcomes in older patients with BC.

## 1 Introduction

Bladder cancer (BC) is predominantly a disease of older adults–over 90% of cases in the United States occur in men above 45 years of age (Godlewski et al., 2024). Advanced age is one of the strongest risk factors for BC incidence and is associated with worse clinical outcomes; patients diagnosed after age 60 have significantly lower long-term survival than younger patients (Lin et al., 2023; Lenis et al., 2020). Several biological phenomena accompanying aging may contribute to this increased risk. Immunosenescence, the age-related decline in immune surveillance coupled with chronic low-grade inflammation (often termed “inflammaging”), is thought to create a pro-tumorigenic milieu in the elderly (Martin et al., 2022). Indeed, older individuals exhibit elevated systemic levels of inflammatory cytokines and an impaired anti-tumor immune response, which can facilitate cancer initiation and progression (Rentschler et al., 2022). At the same time, epigenetic drift, a process by which DNA methylation levels at thousands of CpG sites gradually shift from the pattern seen in young adults, occurs with aging (Issa, 2014). This progressive and stochastic deviation in methylation is driven by imperfect maintenance methylation and long-term environmental exposures. These epigenetic alterations can lead to the silencing of tumor-suppressor genes or activation of oncogenes, essentially “pre-setting” the stage for malignant transformation in aging tissues. Consistent with this, recent evidence indicates that biological age acceleration, as measured by DNA methylation “epigenetic clocks,” correlates with higher BC risk and poorer outcomes independent of chronological age (Deng et al., 2024).

**FIGURE 1:**
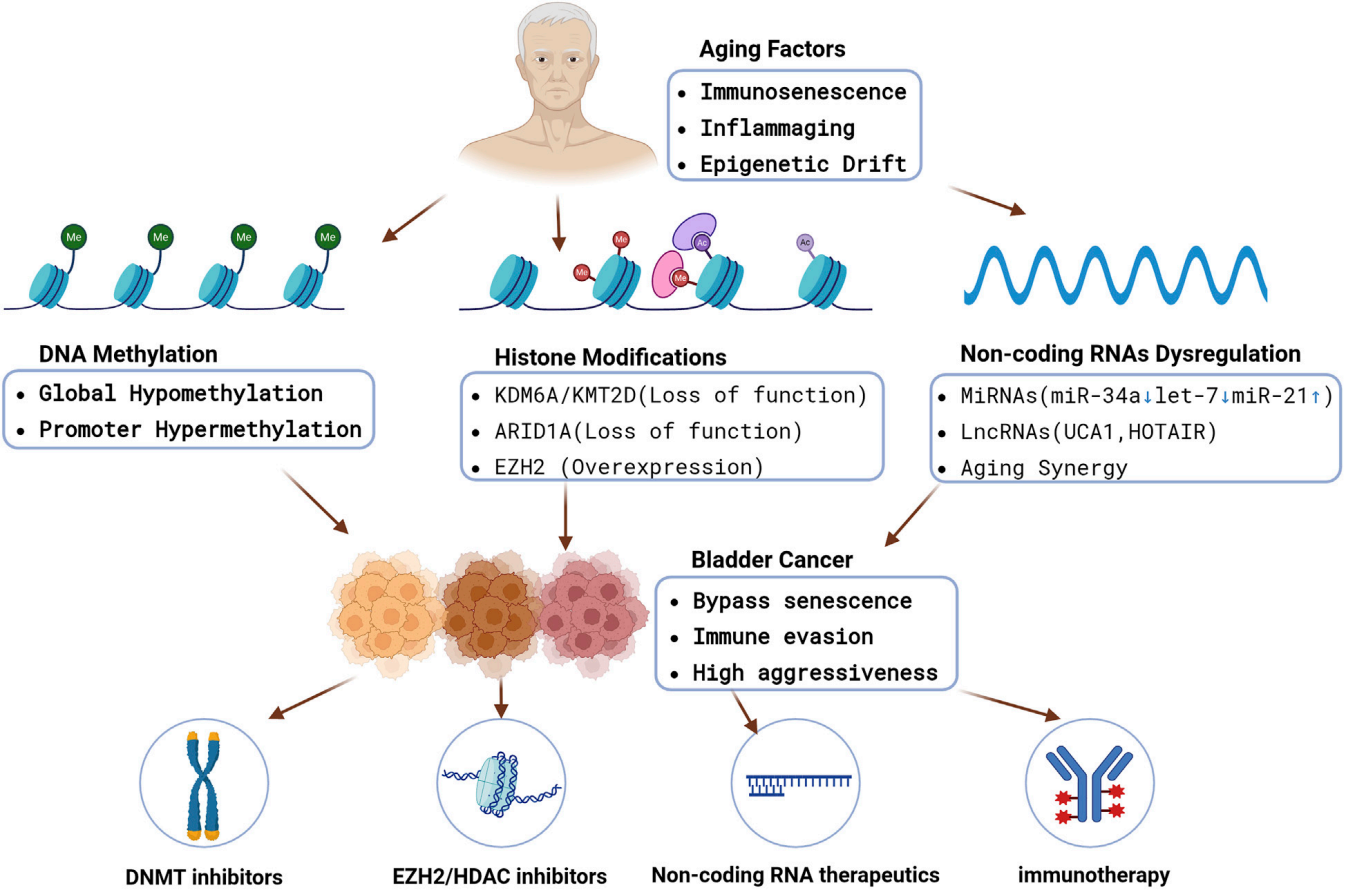
An integrated schematic showing how aging-driven processes (immunosenescence, inflammaging, epigenetic drift) converge on DNA methylation changes, histone modifications, and non-coding RNA dysregulation. These epigenetic alterations collectively promote bladder cancer initiation, immune evasion, and aggressive phenotypes in older patients. Epigenetic therapies (DNMTi, EZH2i, HDACi), non-coding RNA therapeutics, and immunotherapy combinations may reverse key lesions and restore immune surveillance. https://www.biorender.com/.

Critically, many of the molecular hallmarks of aging–such as genomic instability, telomere attrition, and cellular senescence (López-Otín et al., 2013) – are closely interwoven with epigenetic regulatory changes. In bladder urothelium, age-related transcriptional shifts include increased expression of cell-cycle inhibitors (e.g., CDKN2A/p16-INK4A) and pro-inflammatory genes, reflecting an accumulation of senescent cells and inflammatory signals (Evangelou et al., 2023). However, bladder tumors frequently circumvent these aging defenses. For example, the CDKN2A gene (normally upregulated in aging cells to restrain proliferation) is often inactivated by promoter hypermethylation in BC, allowing tumor cells to bypass senescence (Jiao et al., 2018). To understand BC in the context of aging, it is therefore crucial to examine the epigenetic alterations that characterize bladder tumors–including changes in DNA methylation, histone modifications/chromatin remodeling, and non-coding RNA dysregulation alongside age-related processes like immunosenescence and epigenetic drift. Figure 1 provides a schematic overview of the key epigenetic mechanisms driving BC and illustrates how aging may modulate them, ultimately influencing tumor behavior and therapeutic responses in elderly patients. The figure also highlights emerging implications for epigenetic therapies, which may be especially beneficial for older individuals with BC.

## 2 DNA methylation in aging and bladder cancer

Aberrant DNA methylation patterns are a well-established feature of bladder carcinogenesis and may be exacerbated by aging. In normal cells, aging is accompanied by cumulative DNA methylation changes–a phenomenon of epigenetic drift–including global hypomethylation and site-specific hypermethylation at certain gene promoters (Ciccarone et al., 2018). Bladder tumors display these alterations to an extreme degree. On one hand, cancer genomes undergo global hypomethylation with age and tumor progression, which can activate transposable elements and oncogenes, contributing to genomic instability. On the other hand, there is often focal hypermethylation of tumor-suppressor gene promoters in BC, leading to their transcriptional silencing. Researchers have identified hypermethylation in the promoters of over 50 genes in bladder tumors, providing an alternate route to gene inactivation beyond mutations. For instance, the CDKN2A gene (which encodes the p16INK4A protein) is frequently methylated in superficial (early-stage) bladder cancers, an event that likely occurs early in tumorigenesis (Gilyazova, et al., 2023b). This epigenetic silencing of CDKN2A is especially noteworthy in the context of aging: while normal elderly cells rely on CDKN2A upregulation to enforce senescence, bladder tumor cells can epigenetically shut down CDKN2A expression, thus evading an age-imposed growth arrest (Safwan-Zaiter et al., 2022).

Several other tumor suppressors and DNA repair genes are commonly silenced by DNA methylation in bladder cancer. Examples include TP53, RB1, E-cadherin (*CDH1),* among others, which have all been found to be hypermethylated in a subset of bladder tumors (Tran et al., 2021). In addition to these well-known cancer-related genes, other methylation targets such as *SFRP1, LAMC2,* and *SOX9* have been associated with higher tumor grade, stage, and poorer survival in bladder cancer patients (Ye et al., 2021). Collectively, these findings suggest that age-related epigenetic lesions in the tumor (acquired over decades) can drive a more aggressive phenotype. Notably, one study stratified bladder tumors into molecular subgroups based on DNA methylation profiles and found that the subgroup with the highest burden of promoter hypermethylation had the worst prognosis (Luo and Vögeli, 2020). These findings support the concept that an accumulation of DNA methylation abnormalities–which can be viewed as an epigenetic “memory” of aging and carcinogenic exposures–contributes to tumor progression.

Aging not only induces static promoter hyper- and hypomethylation in BC but also reshapes systemic immunity through cumulative DNA-methylation drift captured by “epigenetic clocks.” When methylation-derived biological age is regressed on chronological age, the residual—epigenetic-age acceleration (EAA)—quantifies the speed of epigenetic aging. In 601 non-muscle-invasive BC patients, each 1-year increment in peripheral-blood PhenoAge-EAA raised the 10-year overall-mortality risk by 6% (multivariable-adjusted HR = 1.06, 95% CI 1.03–1.08) and coincided with a neutrophil-rich, memory-T-cell–poor immune profile (Chen et al., 2023b). CpGs that receive high weights in several epigenetic clocks—ranging from five-site forensic models to the PhenoAge and Horvath algorithms—tend to cluster near ELOVL2, FHL2, KLF14, TRIM59, RCAN1, CD46—genes implicated in lipid metabolism, T-cell signaling, and complement control—suggesting a mechanistic bridge between systemic epigenetic aging and impaired anti-tumor immunity (McCartney et al., 2021; Jung et al., 2019). Comparable EAA–prognosis associations have been reported in colorectal, hepatocellular, and breast carcinomas (Horvath, 2013), indicating that systemic epigenetic aging is a pan-cancer modifier whose quantitative impact depends on tumor context. Mechanistically, age-related hypermethylation of cytotoxic T-lymphocyte genes or antigen presentation machinery can blunt immune surveillance (Chen et al., 2023b). Consistent with this, loss of MHC class I expression is frequently observed in recurrent tumors and BCG-unresponsive disease (Wieczorek and Garstka, 2021), and while genetic loss can be one cause, there is evidence that epigenetic mechanisms (like promoter methylation of antigen-processing genes) contribute to immune escape. Indeed, demethylating agents can upregulate MHC and antigen presentation in some cancers, making tumor cells more visible to the immune system (Jongsma et al., 2021). Although this strategy is still under investigation in BC, it underscores how age-associated epigenetic repression of immune-related genes might be reversed for therapeutic benefit.

From a diagnostic and therapeutic standpoint, DNA methylation changes offer attractive biomarkers in the aging population with BC. Methylated DNA can be detected in urine sediments as a noninvasive test, which is especially useful for older patients who may not tolerate frequent invasive cystoscopies. Several urine DNA methylation assays (targeting panels of genes like *GHSR, SST, PRDM14,* and others) have shown high sensitivity for bladder cancer detection (Hentschel et al., 2022). Moreover, epigenetic biomarkers could help predict which older patients are at risk of aggressive disease. For example, an elevated “methylation age” or hypermethylation of certain promoters may indicate tumors with more aggressive behavior, independent of the patient’s chronological age. Clinically, epigenetic therapy using DNMTi is an area of interest. Hypomethylating agents such as decitabine and azacitidine, which are already used in myelodysplastic syndromes (another disease of the elderly) (Zhou et al., 2024), could in theory “rejuvenate” the epigenome of bladder cancer cells–reactivating silenced tumor suppressors and immune genes. While these agents are not yet standard for BC, preclinical studies suggest they can slow tumor growth and enhance immune recognition of bladder tumor cells (Hu et al., 2021; Nunes et al., 2020). Thus, reversing age-related DNA methylation changes holds promise as a therapeutic avenue, particularly for older BC patients who may derive dual benefit from tumor suppression and improved immunogenicity (Pereira et al., 2024).

## 3 Sex-biased epigenetic aging and hormonal crosstalk

Bladder cancer incidence is four times higher in men, yet women who develop the disease often present with more advanced stage and poorer stage-adjusted survival (Doshi et al., 2023). Recent population studies using DNA-methylation “clocks” reveal that peripheral-blood EAA is on average ≈ 1.5 years higher in men than in women, even after correcting for smoking and body-mass index (Kankaanpää et al., 2021). In cancer cohorts, the adverse prognostic impact of high EAA is likewise stronger in males, suggesting that sex modifies the biological consequences of methylation drift (Yu et al., 2021).

A principal driver of this asymmetry is androgen-receptor (AR) signaling. AR recruits DNMT1 and the histone-methyltransferase EZH2 to target promoters, accelerating hyper-methylation and H3K27me3 deposition on tumor-suppressor genes (Zimmerman et al., 2023); pharmacological or genetic AR blockade in the N-butyl-N-(4-hydroxybutyl) -nitrosamine (BBN) mouse model attenuates both methylation drift and tumor incidence (Doshi et al., 2023). Conversely, predominantly via Erβ, estrogen signaling upregulates DNA-repair genes such as BRCA1 and MGMT and slows clock-CpG drift (Zach et al., 2022; Paranjpe et al., 2016); post-menopausal estrogen decline is accompanied by an abrupt 6–8-year jump in the PhenoAge-EAA in large female cohorts (Levine et al., 2016). These hormonal effects intersect with chromatin architecture: AR activation promotes EZH2 overexpression, whereas ERβ can suppress EZH2 transcription, partially explaining the higher prevalence of EZH2-high, immune-cold tumors in older men.

Sex chromosomes add a further layer. KDM6A, an X-chromosome escapee histone-demethylase, is expressed from both alleles in females but from a single allele in males; functional loss abolishes the female survival advantage in murine urothelial carcinogenesis and correlates with reduced CD8^+^ infiltration in human BC(Kaneko and Xue, 2018; Chen et al., 2021a). Because KDM6A counteracts EZH2, its hemizygous state in men may sensitize the male epigenome to AR-EZH2-driven repression, reinforcing the cascade from systemic EAA to local immune evasion (Ramakrishnan et al., 2019; Villanueva, 2017).

Clinical ramifications are two-fold. First, sex-stratified EAA cut-offs could sharpen prognostic models; a 1-year PhenoAge-EAA carries a greater hazard in men than in women. Second, therapy choices might be sex-specific: AR antagonists or EZH2i combined with checkpoint blockade warrant prioritization in EAA-high male patients, whereas estrogen-supplemented PARP- or DNMT-inhibitor regimens could be explored in post-menopausal females with accelerated EAA (Doshi et al., 2023). Taken together, these sexually dimorphic pathways link systemic epigenetic ageing to chromatin-level repression and immune escape—mechanisms that segue directly into the next section on histone modifications and chromatin remodeling.

## 4 Histone modifications and chromatin remodeling in the elderly

Beyond DNA methylation, histone modifications and chromatin remodeling are key epigenetic regulators that are frequently perturbed in BC–often through mutations in histone-modifying enzymes or chromatin regulators. Notably, some of the most commonly mutated genes in bladder tumors encode epigenetic modifiers, indicating how central these epigenetic regulatory processes are to disease pathogenesis (Knowles and Hurst, 2015). For example, the histone methyltransferase gene KMT2D (also known as MLL2) and the histone demethylase gene KDM6A (UTX) are mutated in approximately 20%–30% of bladder cancers, as reported by The Cancer Genome Atlas (TCGA) analyses (Schulz et al., 2019). These loss-of-function mutations have significant epigenetic consequences: KDM6A normally removes repressive H3K27 methylation marks, so its loss can lead to an increase in the repressive H3K27me3 mark on tumor suppressor gene promoters (Tran et al., 2020). Meanwhile, *KMT2D* is an ‘epigenetic writer’ of the activating H3K4me mark; its mutation might reduce expression of genes needed for maintaining differentiated urothelial cell identity (Wang et al., 2025). The net effect of these mutations is an epigenetic shift toward a more primitive, proliferative, and invasive transcriptional program in the cancer cells.

Importantly, the impact of such mutations may be modulated by patient age. Older patients, by virtue of longer exposure to carcinogens and a lifetime of cell divisions, are more likely to accumulate multiple hits in these chromatin regulators. There is also evidence that the consequences of losing certain epigenetic regulators might be more pronounced in the aging context. For instance, ARID1A–a component of the SWI/SNF chromatin remodeling complex–is another frequently mutated gene (∼20% of BC) whose loss can destabilize chromatin structure. In an older cell, which already may have heterochromatin decrease and more transcriptional noise, loss of ARID1A could further promote genomic chaos and dedifferentiation (Zhang et al., 2020; Braga et al., 2020). Moreover, these ARID1A alterations frequently coincide with high tumor mutational burden (TMB) subtypes of bladder cancer (Conde and Frew, 2022). This suggests an interplay in which aging-associated exposures induce these mutations, thereby confer selective advantages in the context of an aged (and perhaps inflammation-rich) tissue microenvironment.

Apart from mutations, dysregulation of histone-modifying enzymes also occurs in bladder cancer and can relate to aging processes. One prominent example is EZH2, the catalytic subunit of Polycomb Repressive Complex 2 (PRC2) that trimethylates histone H3 on lysine 27 (H3K27me3) to silence genes. EZH2 levels increase in many cancers, including aggressive bladder carcinomas (Thompson et al., 2023). Intriguingly, age-related changes in tissue often involve shifts in PRC2 activity–for instance, stem cells in older organisms exhibit altered PRC2 targets - which may help explain why EZH2 is frequently overexpressed in high-grade, muscle-invasive bladder tumors and is correlated with shorter time to recurrence (Piunti et al., 2022). This overexpression might be partly driven by the loss of antagonistic regulators like KDM6A (since KDM6A normally opposes EZH2’s mark) (Taylor-Papadimitriou and Burchell, 2022). Functionally, EZH2 overactivity in tumors leads to silencing of differentiation genes (e.g., E-cadherin) and immune-related genes, contributing to a more malignant and immune-evasive phenotype (Martínez-Fernández et al., 2015b). From the aging perspective, an older patient’s immune system is already less responsive; if a tumor upregulates EZH2 and thereby suppresses antigen presentation or chemokine production, the immunosurveillance might be especially ineffective. Notably, recent studies demonstrated that EZH2-mediated repression plays a direct role in immune escape in bladder cancer. In a carcinogen-induced bladder cancer model, inhibition of EZH2 resulted in significantly reduced tumor progression *only* when the adaptive immune system was intact–in mice lacking T-cells, EZH2 inhibitors had little effect. EZH2 inhibition was found to activate the immune response by upregulating MHC class II and other immune genes in the tumor microenvironment, effectively reversing an epigenetically enforced immunosuppressive state (Piunti et al., 2022). These findings are highly relevant to older patients: they imply that epigenetic therapies targeting repressive histone marks (like H3K27me3 via EZH2i) could rejuvenate anti-tumor immunity, counteracting immunosenescence (Allegra et al., 2023).

Another layer of evidence linking histone modifiers to immune regulation is provided by KDM6A (UTX) mutations. Beyond its general tumor-suppressive role, *KDM6A* loss in BC has been shown to attenuate the anti-tumor immune response. Tumors with KDM6A mutations tend to have fewer tumor-infiltrating lymphocytes (especially CD8^+^ T cells) and a microenvironment skewed toward immune tolerance (Chen et al., 2021a). Transcriptomic analyses indicate that KDM6A-mutant bladder cancers have downregulation of multiple interferon and chemokine signaling pathways required for effective tumor immunity (Kobatake et al., 2020). Clinically, low KDM6A expression in tumors is associated with worse outcomes in BC patients (Alessandrino et al., 2020). One can surmise that in an elderly patient with an already waning immune system, the loss of KDM6A could be a double blow–epigenetically silencing immune-response genes in the tumor and thus further reducing immune cell recruitment to the tumor site. This synergy between aging and epigenetic mutation may partly explain why older BC patients often do not mount strong anti-tumor immune reactions, and why they might respond differently to therapies. It also opens the door to potential combination therapies: for example, using EZH2i or HDACi to epigenetically reprogram “cold” tumors into “hot” (T-cell inflamed) tumors, thereby improving the efficacy of immunotherapies like PD-1 checkpoint inhibitors (Kalbasi and Ribas, 2020). Currently, early-phase clinical trials are exploring this concept–a combination of the EZH2 inhibitor tazemetostat with the PD-1 immunotherapy pembrolizumab was found to be tolerable in advanced urothelial carcinoma, with hints of enhanced immune activation​(Shin et al., 2022). Such epigenetic-immunotherapy combos may be especially relevant for older patients, who often have immunosenescent T cells that need extra stimulation to attack cancer.

In summary, bladder cancer’s alterations in histone modification pathways–whether via mutation (KDM6A, ARID1A, KMT2D) or overexpression (EZH2, HDACs) – are a central component of its biology and interact with aging. The aged epigenome already undergoes shifts like loss of heterochromatin and redistribution of histone marks; bladder tumors build on this by manipulating histone modifiers to promote uncontrolled growth and immune evasion. Recognizing these changes has direct clinical significance. Epidrugs (epigenetic drugs) that target histone-modifying enzymes (e.g., EZH2i, HDACi) could potentially reverse some age-related epigenetic advantages that tumors possess. In preclinical models, targeting these enzymes not only slows tumor proliferation but also unmasks the tumor to the immune system (Yu et al., 2024). Therefore, therapies aimed at the “histone code” may turn out to be particularly impactful in older bladder cancer patients, converting their immunologically silent tumors into ones that can be cleared by the patient’s immune system or by immunotherapy.

## 5 Non-coding RNAs, aging, and bladder cancer

Non-coding RNAs, including miRNAs, lncRNAs, and other types (e.g., circular RNAs), play critical roles in the epigenetic regulation of gene expression (Nemeth et al., 2024). Dysregulation of these RNAs is a hallmark of bladder cancer, and contributes to its development and progression (Zou et al., 2024). Moreover, the expression profiles of certain non-coding RNAs change with age (sometimes called “gero-miRNAs”), which can influence cellular senescence, chronic inflammation, and tumorigenesis (Wagner et al., 2024). In BC, many tumor-suppressive miRNAs are downregulated, while oncogenic miRNAs are upregulated. This imbalance often creates a gene expression pattern that promotes malignancy. Aging can exacerbate these patterns by altering miRNA biogenesis and turnover mechanisms (Sanz-Ros et al., 2023).

### 5.1 MicroRNAs

These short (∼22 nucleotide) RNAs normally fine-tune gene networks post-transcriptionally. In the aging immune system, for example, specific miRNAs like *miR-181a* decline, leading to reduced T-cell sensitivity, whereas others like *miR-146a* increase to suppress chronic inflammation (Kim et al., 2021; Gilyazova et al., 2023a).

In bladder cancer, tumor-suppressive miRNAs that normally inhibit oncogenic pathways are frequently downregulated. One striking example is miR-34a, a miRNA known to be induced by p53 and involved in enforcing cell cycle arrest and senescence. MiR-34a directly targets cell cycle regulators such as CDK6; in aged cells, miR-34a reinforces senescence by restraining proliferation (Ito et al., 2010). However, bladder tumors frequently exhibit downregulation of miR-34a, resulting in upregulation of CDK6 and uncontrolled cell division (Li et al., 2014a). The loss of miR-34a′s restraining influence may be especially detrimental in older patients’ tumors, which often already harbor p53 pathway disruptions. Essentially, a critical cell cycle brake activated during aging is disabled in cancer.

Similarly, *miR-125b*–which suppresses oncogenic transcription factor E2F3 and is associated with cellular aging–is downregulated in bladder cancer, leading to overexpression of E2F3 and accelerated tumor cell proliferation (Wang et al., 2020). *Let-7* family miRNAs, which generally promote differentiation and inhibit proliferation (and tend to increase with cellular senescence), are also commonly diminished in BC, thereby derepressing RAS oncogenes and other targets (Liang et al., 2020; Johnson et al., 2005).

Conversely, certain oncomiRs (oncogenic miRNAs) such as *miR-21* are upregulated in bladder tumors (Ohno et al., 2016); *MiR-21* inhibits apoptosis and is associated with pro-inflammatory senescent secretomes in aged tissues; its overexpression correlates with advanced disease (Syed et al., 2024). Collectively, these miRNA alterations drive a transcriptomic shift toward proliferation, invasion, and apoptosis resistance in the tumor. Aging might facilitate these changes by impairing the miRNA processing machinery (e.g., Dicer and Drosha levels can decline with age) and by chronic inflammatory signals that modulate miRNA expression (Proshkina et al., 2020).

### 5.2 Long non-coding RNAs

LncRNAs (>200 nucleotides) can regulate gene expression through interactions with DNA, RNA, or proteins at both transcriptional and post-transcriptional levels. The bladder cancer transcriptome contains hundreds of abnormally expressed lncRNAs, many of which have important functions in tumor biology (Li et al., 2021). Some lncRNAs act as oncogenes, promoting cell proliferation, metabolic reprogramming, invasion, and therapy resistance, while others function as tumor suppressors.

Aging influences lncRNA expression as well; for example, lncRNAs involved in senescence (e.g., GAS5, a growth-arrest associated lncRNA) tend to accumulate in aged cells but are frequently downregulated in cancers (Li et al., 2020; Wang et al., 2021b). In BC, one of the first identified oncogenic lncRNAs is UCA1 (Urothelial Carcinoma-Associated 1). UCA1 was originally cloned from BC and is overexpressed in bladder tumor tissues (Zhang et al., 2013). It drives tumor progression through multiple mechanisms: UCA1 can act as a molecular sponge for tumor-suppressive miRNAs, it modulates signaling pathways like mTOR and STAT3 to enhance glycolysis and proliferation, and it interacts with epigenetic regulators to alter gene expression (Zhang et al., 2019; Li et al., 2014b). Notably, UCA1 has been shown to sequester miR-143 and miR-145 (both downregulated in aging and cancer), leading to upregulation of metabolic enzymes (HK2) and EMT regulators (ZEB1/2) that drive BC cell invasion (Luo et al., 2017; Xue et al., 2015). The result is a more aggressive tumor phenotype, which may be more common in older patients’ tumors that often exhibit these metabolic and invasive traits.

Another well-studied lncRNA in BC is HOTAIR, which is overexpressed in multiple age-related cancers. HOTAIR originates from the HOX gene cluster and epigenetically silences genes by recruiting PRC2 (EZH2) to specific genomic regions. In bladder cancer, high HOTAIR expression correlates with metastasis and poor clinical outcomes​. Mechanistically, HOTAIR directs EZH2-mediated repression of tumor-suppressive miRNAs (e.g., miR-205) and downstream targets, thereby driving cell cycle dysregulation and invasive behavior (Martínez-Fernández et al., 2015a). Since EZH2 is already elevated in many elderly aggressive tumors, HOTAIR further amplifies its oncogenic effect.

Interestingly, certain lncRNAs are emerging as immune regulators as well. For example, lncRNA LINC00337 (Lnc-LBCS) has been reported to suppress bladder cancer “stemness” by binding to the chromatin modifier EZH2 and the RNA-binding protein hnRNPK, forming a complex that represses the stem cell gene SOX2 (Chen et al., 2019). Such lncRNAs might actually support immune function by keeping tumor cells differentiated and more recognizable to the immune system. Age-related decline in these protective lncRNAs could predispose elderly BC patients to tumors with stem-like properties and immune evasion capabilities.

Overall, non-coding RNAs form a critical bridge between aging and cancer. In aging tissues, the balanced expression of miRNAs and lncRNAs helps maintain homeostasis–for instance, by eliminating senescent cells and modulating inflammation. When this balance is disrupted (either by age-related dysregulation or genetic/epigenetic changes in a tumor), the result can be unchecked cell growth and a tumor-promoting microenvironment. Bladder cancer exploits this by downregulating miRNAs that would normally enforce senescence or apoptosis, and upregulating lncRNAs that drive proliferation and metastasis. Clinically, these molecules hold promise as biomarkers and therapeutic targets, particularly in older patients who may face challenges with traditional interventions. Urine-based miRNA signatures (e.g., miR-21, miR-141, and miR-205 panels) have shown potential for non-invasive bladder cancer detection (Ghorbanmehr et al., 2019; Chattopadhaya et al., 2025). Likewise, lncRNA expression profiles can predict tumor behavior; for example, an eight-long lncRNA signature was reported to predict recurrence in BC (Lian et al., 2019). Future therapies might include miRNA mimics or inhibitors to restore a youthful, tumor-suppressive miRNA environment, or antisense oligonucleotides (ASOs) targeting oncogenic lncRNAs like UCA1 and HOTAIR. Such strategies could complement existing treatments, possibly resensitizing tumors to chemotherapy or immunotherapy in elderly patients.

A concise overview of these key epigenetic alterations, their links to aging, and their impact on BC progression is provided in Table 1.

**TABLE 1:** Key Epigenetic Alterations in Bladder Cancer and Their Links to Aging. This table summarizes major epigenetic regulators, their age-associated changes, and how they drive bladder tumor progression.

Epigenetic Factor	Connection to Aging	Impact on Bladder Cancer	References
CDKN2A (p16-INK4A)	Upregulated in aged cells to enforce senescence	Downregulation bypasses senescence and promotes uncontrolled proliferation	Jiao et al. (2018) Safwan-Zaiter et al. (2022)
KMT2D (MLL2)	Mutations accumulate with age, impairing normal urothelial differentiation	Reduces expression of differentiation genes and drives more aggressive phenotypes	Wang et al. (2025)
KDM6A (UTX)	Age-associated mutations lead to excessive repressive chromatin marks	Silences tumor suppressors and immune genes, enhancing immune evasion	Schulz et al. (2019) Chen et al. (2021)
ARID1A	Loss of function co-occurs with genomic instability in aged cells	Promotes dedifferentiation and is linked to high-risk bladder cancer	Zhang et al. (2020) Braga et al. (2020)
EZH2	Overexpressed in elderly aggressive tumors	Represses genes for differentiation and immune response	Martínez-Fernández et al. (2015b)
miR-34a	Declines with aging, partly due to reduced miRNA processing	Loses tumor-suppressive control on cell cycle, leading to proliferation	Ito et al. (2010) Li et al. (2014a)
let-7	Increases with cellular aging, restricting oncogene expression	Downregulation derepresses RAS and accelerates tumor progression	Liang et al. (2020) Johnson et al. (2005)
miR-21	Associated with chronic inflammation in aged tissues	Inhibits pro-apoptotic genes, fueling advanced disease	Ohno et al. (2016) Syed et al. (2024)
UCA1	Overexpressed in elderly patients with chronic inflammation	Enhances EMT, metabolic reprogramming, and therapy resistance	Zhang et al. (2019) Li et al. (2014b)
HOTAIR	Upregulated in multiple age-related cancers	Drives metastasis and cell-cycle dysregulation	Martínez-Fernández et al. (2015a)

## 6 Conclusion and future directions

Aging profoundly shapes the epigenetic landscape of bladder cancer. As outlined, older patients with BC often exhibit tumors with widespread DNA methylation abnormalities, mutations in chromatin-modifying genes, and altered non-coding RNA profiles. These changes are not merely passive bystanders but actively drive tumor initiation, progression, and immune evasion. An emerging theme is that bladder tumors effectively co-opt age-related epigenetic processes to their advantage–for instance, silencing senescence regulators (such as p16-INK4A and *miR-34a)*, upregulating immune-suppressive factors (like EZH2 and HOTAIR), and exploiting the chronic inflammatory microenvironment of aged tissues (Balaraman et al., 2025; Nutt et al., 2020). Understanding these interactions between aging and epigenetics opens up new avenues to improve management of BC in older adults. Key future directions include the following.

### 6.1 Epigenetic biomarkers of aging

The development of methylation- and non-coding-RNA-based biomarkers capable of gauging the “biological age” of bladder tumors has emerged as an important research focus. Some studies suggest that these biomarkers may stratify patients by tumor aggressiveness and predict therapeutic response (Horvath, 2013). For example, an epigenetic clock signature derived from tumor or blood DNA could help identify older patients whose cancers are biologically more aggressive (Wang et al., 2021; Chen et al., 2022). Such patients might benefit from earlier aggressive treatment or epigenetic therapy, such as immune checkpoint inhibitors (e.g., anti-PD-1 or anti-PD-L1 therapies) or targeted epigenetic therapies, including DNA methyltransferase inhibitors (e.g., azacitidine or decitabine). Conversely, detecting particular methylation patterns (e.g., a panel of hypermethylated genes such as *CDKN2A* or *RASSF1A*) in urine samples could enable non-invasive early detection of BC in the elderly, when tumors are still localized and treatable (Ibrahim et al., 2023; Lin et al., 2010).

### 6.2 Microbiome–epigenetic interactions in the ageing host

Accumulating evidence indicates that age-related dysbiosis of the gut microbiome can reprogram the bladder epigenome through metabolite-mediated crosstalk. Metagenomic surveys show that bladder cancer patients harbor fewer butyrate-producing taxa such as Lachnospiraceae and Prevotella, accompanied by a measurable drop in fecal butyric-acid concentrations and intestinal-barrier integrity. Butyrate and other short-chain fatty acids (SCFAs) act as endogenous class I/IIa HDACi; their depletion removes a physiological brake on HDAC activity, thereby favoring re-establishment of repressive chromatin and promoter hypermethylation at tumor-suppressor loci (e.g., CDKN2A) in aging urothelium (Schilderink et al., 2013; Borrego-Ruiz and Borrego, 2024; Chen et al., 2023a). Therapeutic corollary: restoring SCFA-producing consortia (high-fiber diets, Faecalibacterium or Akkermansia probiotics) or delivering intravesical butyrate analogs may synergize with DNMTi, HDACi or EZH2i to resensitize “cold” tumors in older patients.

### 6.3 Targeted epigenetic therapies

Targeted epigenetic therapies that seek to reverse pathogenic chromatin marks—particularly in immunosenescent patients—are now being actively explored as a novel treatment modality for BC. A recent review identified dozens of clinical trials evaluating epigenetic therapies in BC, including DNMTi (e.g., 5-azacytidine and decitabine), HDACi, and EZH2i (Thompson et al., 2023). While results have been mixed and no phase III trial has yet been completed, epigenetic drugs have shown potential synergy with existing treatments. For instance, demethylating agents may increase tumor immunogenicity and improve responses to immunotherapy. The data from preclinical models are encouraging–for instance, DNMTi and HDACi essentially “flip the switch” on an immune-cold tumor to make it immune-hot (Galustjan 2023), an effect that could synergize with checkpoint blockade. For older patients who often have attenuated immune function, this could be transformative. Another promising strategy involves targeting telomere epigenetics. Aging-associated telomere shortening and dysregulation of telomere-associated lncRNAs (e.g., TERRA) drive genomic instability in cancer (Chu et al., 2017). Recent studies suggest that modulating telomeric chromatin states (e.g., restoring heterochromatin marks like H3K9me3) could selectively trigger tumor cell crisis (via mitotic catastrophe) while sparing normal aged cells (Cacchione et al., 2019).

### 6.4 Delivery challenges and emerging solutions

Systemic administration of first-generation epidrugs such as azacitidine and decitabine inevitably exposes healthy proliferative tissues to genome-wide demethylation, leading to myelosuppression, gastrointestinal toxicity and, paradoxically, a potential pro-tumorigenic milieu (Zhou et al., 2024). To mitigate these off-target effects, intravesical delivery—already standard for BCG and gemcitabine—has been repurposed for epigenetic agents: weekly bladder instillation of azacitidine in a carcinogen-induced mouse model delayed tumor onset and prolonged survival without the marrow toxicity observed after equivalent intravenous dosing (Wang S. -C. et al., 2021). Building on this concept, cationic liposomes, polymeric nanoparticles and hydrogel depots that penetrate the glycosaminoglycan layer can maintain therapeutic drug levels at the urothelial surface for days, convert “cold” tumors to “hot” phenotypes and spare hematopoietic cells in xenografts (Zhao et al., 2024; Wang et al., 2023). Collectively, these advances suggest that rational drug-delivery engineering, aligned with bladder-specific anatomy, could unlock the full therapeutic potential of epidrugs while reducing age-related toxicity.

### 6.5 Combination strategies

Given the interdependence of epigenetic dysregulation and immunosenescence, combination therapies are particularly promising. For example, combining transurethral resection of the bladder tumor (TURBT) with *Bacillus* Calmette-Guérin (BCG) immunotherapy might enhance T-cell recognition of tumor-associated antigens in older adults with non-muscle-invasive bladder cancer (NMIBC) (Lodewijk et al., 2021). Likewise, pairing HDAC or EZH2 inhibitors with PD-1/PD-L1 inhibitors could reverse adaptive immune resistance in advanced malignancies (Kang et al., 2020). Several prospective trials are evaluating combinations of epidrugs with immune-checkpoint inhibitors. Table 2 summaries the key ongoing studies, focusing on trials that are currently recruiting or listed as active but not yet reporting final results. Future clinical trials should prioritize broad age inclusion and conduct age-stratified analyses to determine whether older patients derive unique therapeutic benefits from epigenetic combination therapies.

**TABLE 2:** Clinical Trials of Epigenetic Drug-Immunotherapy Combinations in Bladder Cancer/Urothelial Carcinoma. This table reflects the latest ClinicalTrials.gov update and lists interventional studies that are actively accruing or in follow-up as of June 2025.

NCT ID	Phase	Epigenetic Drug	Immunotherapy	Status & Update Date	Primary Completion
NCT05154994	Ⅰ	Belinostat (HDACi)	Durvalumab	Recruiting (2025-04-24)	2026-04-01
NCT06022757	Ⅰ/Ⅱ	XNW5004(EZH2i)	KEYTRUDA® (pembrolizumab)	Recruiting (2024-02-23)	2028-08

### 6.6 Addressing epigenetic side effects

Safety and tolerability are critical considerations for older patients receiving epigenetic therapies. Epigenetic drugs can have off-target effects, such as myelosuppression (e.g., anemia, thrombocytopenia) or neurocognitive disturbances, due to their broad impact on chromatin remodeling. For example, DNA hypomethylating agents (e.g., azacitidine) are associated with hematologic toxicity, while HDACi can induce myelosuppression and fatigue in older adults (Sun et al., 2018; Navada and Silverman, 2017). To address this, dose optimization based on frailty status and real-time monitoring of blood counts and cognitive function are essential when developing epidrugs for bladder cancer. Notably, Tazemetostat, an oral EZH2 inhibitor, has demonstrated favorable tolerability in older patients with advanced solid tumors, including those with genitourinary malignancies (e.g., prostate cancer) (Izutsu et al., 2021). While direct data in BC are limited, its safety profile and epigenetic mechanism support further exploration in urothelial carcinoma. Future strategies should integrate biomarker-guided personalization to balance efficacy and toxicity.

### 6.7 Epigenome editing

CRISPR/dCas9-based tools enable locus-specific correction of bladder cancer-associated epigenetic aberrations, offering a targeted therapeutic strategy. In some researches, CRISPR-dCas9-VPR was utilized to target the ERIC locus, revealing that its overexpression in T24 and 5637 BC cells significantly suppressed proliferation and invasiveness while promoting apoptosis. Conversely, CRISPR-dCas9-KRAB-mediated knockdown of CacyBP in the same cell lines inhibited proliferation and migration and enhanced caspase-3-dependent apoptosis. These findings underscore the dual utility of epigenome editing—activating tumor suppressors (e.g., ERIC) or silencing oncogenes (e.g., CacyBP)—to reverse malignant phenotypes, offering a precision-based strategy to counteract age-related epigenetic dysregulation (Yang et al., 2021; Zheng and Chen, 2021). In an aging context, such strategies might extend to preventive applications–for example, using dietary or pharmacologic interventions to slow epigenetic drift (as measured by epigenetic age) and thereby reduce BC incidence (Lin and Wagner, 2015). Although currently speculative, these concepts underscore the potential of intervening on the epigenetic level to mitigate age-related cancer risk.

Several epigenetic therapeutics have been investigated in BC, including DNMTi, HDACi, and EZH2i, each holding particular promise in older patients (see Table 3 for mechanisms and clinical relevance).

**TABLE 3:** Overview of Epigenetic Therapeutic Strategies for Bladder Cancer in Elderly Patients. This table highlights current and emerging epigenetic-based treatments, their mechanisms, and clinical relevance for older adults.

Therapy	Mechanism	Clinical Relevance for Elderly BC Patients	Reference
DNMT Inhibitors	Inhibit DNA methyltransferases, reactivate silenced TSGs	Potential to restore senescence pathways and enhance immunogenicity	Nunes et al. (2020)
EZH2 Inhibitors	Block H3K27me3 to reduce gene silencing	May convert immune-cold tumors to immune-hot	Shin et al. (2022)
HDAC Inhibitors	Maintain histone acetylation, reopen chromatin	Could overcome age-related repression and synergy with immunotherapy	Allegra et al. (2023)
Non-coding RNA Therapeutics	Use miRNA mimics or antisense lncRNA to correct dysregulation	Can restore miR-34a or silence UCA1/HOTAIR	X. Zhang et al. (2019) Li et al. (2014b)
CRISPR/dCas9 Epigenome Editing	Precisely activate or repress specific loci	Experimental, potential to reverse age-driven epigenetic drift	Yang et al. (2021) Zheng and Chen (2021)

In conclusion, bladder cancer exemplifies the profound interconnection between cancer biology and aging processes. Epigenetic regulation sits at this nexus, influencing virtually every step from tumor cell-intrinsic behavior to tumor-immune system interactions. By advancing our understanding of epigenetic alterations in the context of aging, we can improve risk prediction, personalize therapeutic strategies, and develop novel therapies for the growing population of older BC patients. Future advancements in BC management may hinge on dual-targeting approaches that simultaneously eliminate malignant cells and restore youthful epigenetic regulation in aged tumor microenvironments and immune systems–potentially resetting dysregulated epigenetic programs to reestablish antitumor responsiveness.
